# Pain perception and treatment time of chemo mechanical versus conventional caries removal techniques in pediatric dental patients

**DOI:** 10.6026/973206300222300

**Published:** 2026-04-30

**Authors:** Mohd Zeeshan Ahmad, Sulakshana Pradeep, Apeksha Ghatge, Shumayla Khan, Brij Kumar, Mary Grace, Pratik Surana

**Affiliations:** 1 Department of Conservative Dentistry & Endodontics, Rama Dental College Hospital & Research Centre, Kanpur, Uttar Pradesh, India; 2 Department of Dentistry, Belagavi Institute of Medical Sciences, Belagavi, Karnataka, India; 3 Department of Pharmacology, Yogita Dental College, Khed, Maharashtra, India; 4 Department of Pediatric and Preventive Dentistry, ITS Dental College, Greater Noida 47, Knowledge Park III, Greater Noida, Uttar Pradesh- 201310, India; 5 Department of Paediatric and Preventive Dentistry, Rungta College of Dental Sciences and Research, Bhilai, Chhattisgarh, India; 6 Private Practitioner, Pediatric Dentist, Balaam Dental and Medical Clinic, Chennai, Tamil Nadu, India; 7 Department of Pedodontics and Preventive Dentistry, Maitri College of Dentistry and Research Centre, Durg, Chhattisgarh, India

**Keywords:** Carie-solve, chemo mechanical caries removal, pediatric dentistry

## Abstract

Pain and fear associated with rotary instruments remain a challenge in pediatric caries management. Hence, this randomized controlled clinical trial included 30 children aged 6-10 years requiring restoration in primary mandibular molars. Caries removal was performed using an airotor or Carie-solve, with pain assessed using the verbal pain scale and treatment time recorded using a stopwatch. Carie-solve showed significantly lower pain scores, while the conventional technique required significantly less time (p = 0.0001). Carie-solve may serve as a minimally invasive, child-friendly alternative for caries removal in pediatric patients.

## Background:

Dental caries remains one of the most prevalent chronic diseases worldwide. During much of the twentieth century, operative dentistry was strongly influenced by the principles proposed by Dr. G. V. Black, particularly the concept of extension for prevention [1]. This philosophy assumed that once initiated, carious lesions would inevitably progress, leading to a predominantly surgical approach to treatment rather than a biological or therapeutic one [2]. Conventional caries excavation involves the use of hand instruments in combination with high- and low-speed rotary handpieces fitted with stainless steel, carbide, or diamond burs. While these techniques are efficient for the rapid removal of carious enamel and dentin, they present several disadvantages [3]. Unnecessary removal of healthy tooth structure, limited tissue preservation and the generation of heat and pressure may compromise pulpal health [4]. In addition, vibration, noise, pain and the frequent requirement for local anesthesia often leads to unpleasant clinical experiences, contributing to fear and anxiety, particularly among pediatric patients [5].

Consequently, there is an increasing demand for minimally invasive caries removal methods that are more comfortable for children while effectively eliminating infected dentin [6]. Over the past decade, chemomechanical caries removal (CMCR) has emerged as one of the most widely adopted modalities within minimally invasive dentistry [7]. This technique involves chemical softening of the carious dentin followed by its gentle removal using hand instruments [8]. Unlike conventional surgical methods, CMCR selectively eliminates infected dentin while preserving affected dentin with remineralization potential, thereby reducing unnecessary tooth structure loss and making the procedure less destructive [9]. Sodium hypochlorite (5%) was initially introduced for chemomechanical caries removal by Habib *et al.* [10] followed by the development of several CMCR agents. GK-101 (Caridex), introduced by Goldman and Kronman was approved by the Federation Dentaire Inter-nationale in the United States in 1984 [11]. Subsequently, Carisolv was developed in Sweden in 1998, Papacarie® in Brazil and Carie-Care emerged as a widely used CMCR agent in India in 2012. More recently, Carie-solve, an enzyme-based CMCR agent, have gained attention due to its papaya-derived active component [12]. As chemomechanical caries removal techniques have the potential to reduce pain and discomfort associated with conventional methods [13, 14]. Therefore, it is of interest to show and compare chemomechanical caries removal with conventional caries removal techniques.

## Materials and Methods:

## Study design:

This study was designed as a randomized controlled clinical trial with a parallel-group design to compare chemomechanical caries removal and conventional caries removal techniques in pediatric patients. The study protocol was approved by the Institutional Ethical Committee and written informed consent was obtained from the parents or guardians of all participating children.

## Study population:

A total of 30 children aged 6-10 years were selected from the outpatient department of Pediatric Dentistry. Children requiring restorative treatment in primary mandibular molars were screened for eligibility.

## Inclusion criteria:

Children aged between 6 and 10 years with dentinal caries in primary mandibular molars requiring restoration were included in the study. Only cooperative children with a Frankl behavior rating of 3 or 4 and teeth without signs of pulpal involvement were selected.

## Exclusion criteria:

Teeth showing pulpal exposure, abscess, or fistula were excluded. Children with systemic illness, special health care needs, or a history of allergy to any component of the chemomechanical agent were also excluded. Uncooperative children were not included in the study. Eligible participants were randomly assigned to two groups using a simple randomization method and allocation was performed immediately before the procedure to minimize selection bias. Group I served as the control group and underwent conventional caries removal using an airotor, whereas Group II served as the experimental group and received chemomechanical caries removal using Carie-solve.

## Conventional caries removal:

Caries excavation was carried out using a large round bur at slow speed. Local anesthesia was not administered to ensure uniform conditions for pain assessment. The cavity was subsequently restored with Type II glass ionomer cement (GC Fuji II), with isolation maintained using cotton rolls.

## Chemo mechanical caries removal:

Caries removal was performed using Carie-solve according to the manufacturer's instructions. Tooth isolation was achieved using cotton rolls, following which the Carie-solve gel was applied to completely cover the cavity for 30-40 seconds. The softened dentin was gently removed using a small spoon excavator with minimal pressure in a pendulum motion. Residual gel was cleaned using a saline-moistened cotton pellet and the procedure was repeated until light brown dentin was visible at the cavity base. The cavity was subsequently restored with Type II glass ionomer cement.

## Pain assessment:

Pain perception was assessed using a verbal pain scale ranging from 0 to 4, where a score of 0 indicated no pain, 1 indicated mild pain that was recognizable without discomfort, 2 represented moderate pain that was discomforting but bearable, 3 denoted severe pain causing considerable discomfort and difficulty in tolerating the procedure and 4 indicated very severe pain [13].

## Time assessment:

The time required for caries removal was measured using a stopwatch, starting from the initiation of caries excavation until complete removal of infected dentin. Time spent on restoration was excluded.

## Statistical analysis:

The collected data were tabulated and statistically analyzed using appropriate software. Pain scores and treatment time between the two groups were compared using non-parametric statistical tests. A p-value of <0.05 was considered statistically significant.

## Results and Discussion:

All 30 children successfully completed the study and data from all participants were included in the final analysis. Pain perception, assessed using the verbal pain scale, was significantly lower in the Carie-solve group compared to the conventional caries removal group, indicating better patient comfort during the procedure. The mean verbal pain score recorded for the Carie-solve group was 1.00 ± 0.65, whereas the conventional airotor group exhibited a significantly higher mean score of 3.80 ± 0.82 (Table 1 - see PDF). Statistical analysis demonstrated a highly significant difference between the two groups (p = 0.0001). With respect to procedural efficiency, the mean time required for caries removal was significantly shorter using the conventional rotary technique (95.20 ± 4.85 seconds) compared to chemo-mechanical caries removal with Carie-solve (187.40 ± 5.60 seconds) (Table 2 - see PDF). The results of our study showed that using Carie-solve reduces discomfort but takes more time (Table 3 - see PDF). Pain and anxiety associated with conventional rotary instruments remain major challenges in pediatric dental care and often influence a child's cooperation and future dental attitude [14]. Conventional caries removal and cavity preparation rely on high-speed handpieces and burs, which enhance the speed and efficiency of treatment; however, these techniques are associated with several inherent drawbacks, including patient discomfort, the frequent need for local anesthesia, thermal and pressure effects on the dental pulp and the inadvertent removal of healthy dentin, leading to excessive loss of sound tooth structure [15]. In the pursuit of newer technologies for caries management, several alternative methods have been introduced. Chemomechanical caries removal has emerged as a minimally invasive, noninvasive approach that selectively eliminates infected dentin while reducing the need for local anesthesia, minimizing pulpal irritation and improving patient comfort [16, 17]. The present randomized controlled clinical trial compared chemomechanical caries removal using Carie-solve with conventional rotary caries removal in children aged 6-10 years, focusing on pain perception and treatment time. The results of this study demonstrated that chemomechanical caries removal using Carie-solve resulted in significantly lower pain scores compared to the conventional airotor technique. The reduced pain perception observed with Carie-solve can be attributed to the absence of rotary instrumentation, vibration, noise and thermal effects, all of which are commonly associated with discomfort during conventional caries excavation. Additionally, the selective action of chemomechanical agents on infected dentin allows gentle removal using hand instruments, thereby minimizing pressure on the pulp and surrounding tissues. The results of the present study are consistent with previous research highlighting the benefits of selective caries removal. Kavvadia and co researchers demonstrated that removal of carious dentin generally produces minimal or no pain, whereas cutting sound dentin often results in increased sensitivity [16]. Similar findings were reported by Zinck *et al.* who suggested that patient discomfort during caries removal is largely associated with the removal of healthy dentin [17]. Conventional rotary instruments, although widely used, are frequently associated with pain and discomfort due to vibration, noise, mechanical pressure on the tooth, thermal stimulation and pulpal sensitivity during cavity preparation [13]. Chemomechanical caries removal systems such as Carisolv act selectively on denatured collagen fibers in demineralized dentin, preserving sound tooth structure and reducing pain; Hegde and Abhishek further reported that this selective removal decreases noise, pain and anxiety particularly in children and anxious or medically compromised patients without adversely affecting healthy dentin or pulpal tissues [18]. Despite the advantage of reduced pain, the present study demonstrated that chemomechanical caries removal using Carie-solve required significantly more time than the conventional technique. The increased treatment duration may be attributed to the repeated application of the gel and the gradual removal of softened infected dentin to achieve complete excavation. Similar observations have been reported by Ansari *et al.* [19] Rafique *et al.* [20] and Chowdhry *et al.* [21] who also noted longer operative times with chemomechanical and minimally invasive techniques compared to conventional rotary methods. Nevertheless, these studies emphasized that the additional time is clinically acceptable when weighed against the benefits of improved patient comfort, reduced anxiety and decreased need for local anesthesia, particularly in pediatric and anxious patients [22]. The findings of this study support the concept of minimally invasive dentistry, which emphasizes the preservation of healthy tooth structure. Chemomechanical caries removal selectively eliminates infected dentin while preserving affected dentin with remineralization potential, thereby reducing unnecessary tooth structure loss [23]. This conservative approach is particularly beneficial in pediatric dentistry, where maintaining tooth integrity is essential for the longevity of primary teeth and proper arch development [24]. However, certain limitations should be considered while interpreting the results. The relatively small sample size and short-term evaluation limit the generalizability of the findings. In addition, the study assessed only pain perception and treatment time; long-term outcomes such as restoration longevity and patient satisfaction were not evaluated. Future studies with larger sample sizes and long-term follow-up are recommended to further validate the clinical effectiveness of chemomechanical caries removal techniques. Overall, within the limitations of the present study, chemomechanical caries removal using Carie-solve appears to be a promising alternative to conventional caries removal, particularly in anxious and pediatric patients, as it significantly reduces pain and discomfort while maintaining a conservative approach to caries management.

## Conclusion:

We report that chemomechanical caries removal chemomechanical caries removal using Carie-solve significantly reduced pain perception compared to conventional rotary caries removal, although it required a longer treatment time. Carie-solve may be considered a safe, minimally invasive and child-friendly alternative for effective caries management in pediatric patients.

## Advancement to knowledge:

This study advances knowledge by demonstrating that chemomechanical caries removal significantly reduces pain perception in pediatric patients, improving comfort and cooperation compared to conventional drilling techniques. However, it also highlights that CMCR requires longer treatment time, emphasizing a trade-off between patient comfort and clinical efficiency.

## References

[1] Chavhan PD (2024). J Clin and Diagn Res..

[2] Philip N, Suneja B (2023). J Conserv Dent..

[3] Goomer P (2013). J Int Oral Health..

[4] Krishnaveni L (2025). Int J Clin Pediatr Dent..

[5] Montedori A (2016). Cochrane Database Syst Rev..

[6] Goyal A (2022). J South Asian Assoc Pediatr Dent..

[7] Mm J (2014). J Clin Diagn Res..

[8] Hamama H (2014). Aust Dent J..

[9] Soni HK (2015). Eur Arch Paediatr Dent..

[10] Habib CM (1975). Pharmacol. Ther. Dent..

[11] Goldman M, Kronman JH (1976). J Am Dent Assoc..

[12] Dadpe MV (2025). Dent Res J (Isfahan)..

[13] Kochhar GK (2011). J Clin Pediatr Dent..

[14] Alia S (2020). Int J Clin Pediatr Dent..

[15] Bussadori SK (2005). J Clin Pediatr Dent..

[16] Kavvadia K (2004). Pediatr Dent..

[17] Zinck JH (1988). J Oral Rehabil..

[18] Hedge MN, Abhishek M (2017). Adv Res Gastroentero Hepatol..

[19] Ansari G (2003). J Oral Rehabil..

[20] Rafique S (2003). Caries Res..

[21] Chowdhry S (2015). Int J Clin Pediatr Dent..

[22] Carmona-Santamaría M (2026). J Clin Med..

[23] Maashi M (2025). Eur Arch Paediatr Dent..

[24] Aydogdu AB (2025). Int J Clin Pediatr Dent..

